# Alteration of Epigenetic Regulation by Long Noncoding RNAs in Cancer

**DOI:** 10.3390/ijms19020570

**Published:** 2018-02-14

**Authors:** Mariangela Morlando, Alessandro Fatica

**Affiliations:** Department of Biology and Biotechnology “Charles Darwin”, Sapienza University of Rome, 00185 Rome, Italy

**Keywords:** lncRNA, epigenetics, chromatin, cancer

## Abstract

Long noncoding RNAs (lncRNAs) are important regulators of the epigenetic status of the human genome. Besides their participation to normal physiology, lncRNA expression and function have been already associated to many diseases, including cancer. By interacting with epigenetic regulators and by controlling chromatin topology, their misregulation may result in an aberrant regulation of gene expression that may contribute to tumorigenesis. Here, we review the functional role and mechanisms of action of lncRNAs implicated in the aberrant epigenetic regulation that has characterized cancer development and progression.

## 1. Introduction

Long noncoding RNAs (lncRNAs) are generally defined as transcripts longer than 200 nucleotides lacking protein coding potential and transcribed by the RNA polymerase II (RNA Pol II) (reviewed in [1]). LncRNAs not overlapping annotated coding genes are generally defined as long intergenic noncoding RNAs (lincRNAs). Here, we will generally refer to lncRNAs for both species. The human genome contains 15,778 lncRNA genes producing 27,908 lncRNA transcripts (Gencode v27 annotation, January 2017), and their number keeps increasing year after year. LncRNAs are uniquely expressed in specific cell types to a greater degree than protein coding RNAs (mRNAs) and they also show specific expression in different cancer types.

Although the proportion of functional lncRNAs is still not clear, many lncRNAs play important regulatory roles in diverse biological processes and their misregulation contributes to different human diseases, including cancer. LncRNAs are heterogeneous in their mechanisms of action, depending on their cellular localization and interacting molecules [1]. In some cases, multiple functional mechanisms in different cellular compartments have been assigned to single lncRNA species.

**Figure 1:**
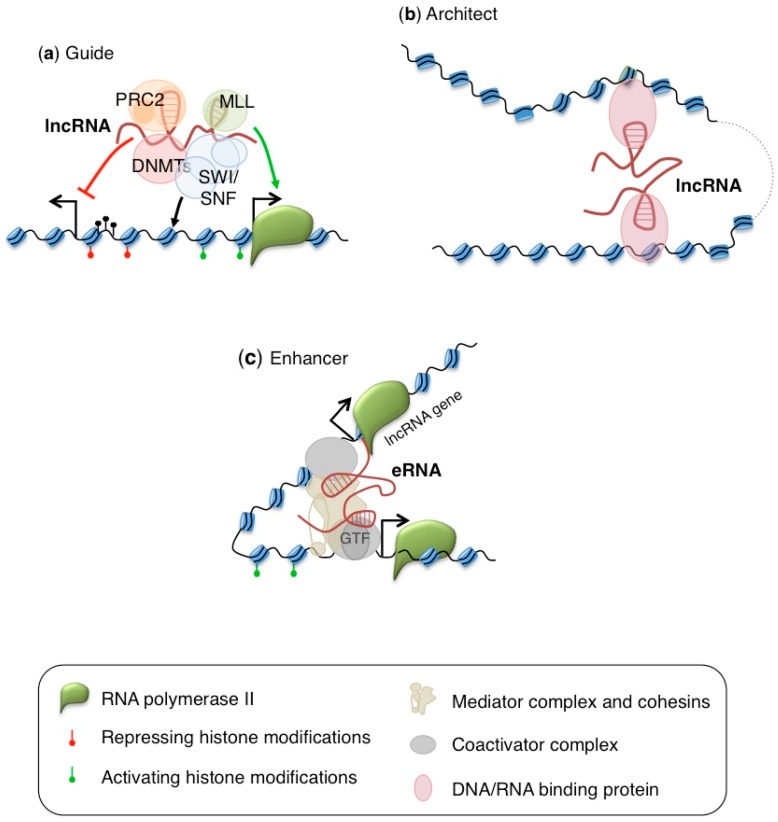
Nuclear lncRNAs may act as: (**a**) guide lncRNAs, which act by recruiting or rejecting epigenetic regulators (chromatin modifying complexes and chromatin remodeling complexes) onto specific chromosomal loci; (**b**) architect lncRNAs, which act by modifying the three dimensional conformation of chromatin; and (**c**) enhancer lncRNAs (also called eRNAs), which regulate transcription through enhancer-like functions.

Perturbations of epigenetic regulation are thought to be a key feature of many cancers, and it is now clear that epigenetic changes drive cancer development [2]. In the nucleus, different lncRNAs act by regulating the epigenetic status of protein-coding genes. These lncRNAs act by guiding epigenetic regulators to specific loci or by orchestrating chromatin folding and compartmentalization to direct enhancer-promoter communication (Figure 1). Nuclear lncRNAs often functionally operate in cis to modify local gene transcription. Cis-acting lncRNAs are often expressed at low abundance, at only a few copies per cell, and their importance might be dismissed or underestimated by high-throughput studies. Some other nuclear lnRNAs act in trans to regulated expression of loci distant from their sites of synthesis or located in different chromosomes. However, how the specific localization of nuclear lncRNAs is achieved and regulated remains largely unknown.

Recent genome-wide approaches have highlighted that mutations in regulatory regions, altering the enhancer and promoter sequences or their chromatin state, lead to abnormal expression of lncRNAs in tumors with respect to the normal tissue counterpart [3,4,5,6]. Misregulated lncRNAs may have a significant impact in different pathological steps of tumorigenesis from proliferation to resistance to apoptosis, angiogenesis and metastasis [7].

In this review, we describe cancer-related lncRNAs directing epigenetic changes at the chromatin level in terms of histone modifications, DNA methylation and chromatin architecture (Table 1), and we discuss their contribution to cancer development.

## 2. Regulators of Histone Marks Deposition

LncRNAs can increase or repress transcriptional activity by controlling the deposition of histone marks on chromatin regions (Figure 1). These lncRNAs function by interacting with chromatin modifier proteins (e.g., methyltransferases, demethylases, acetyltransferases and deacethylases) to promote the formation of macromolecular complexes on specific genomic loci. An important feature is that they often interact with different proteins allowing the coordination of distinct epigenetic regulatory complexes. Their scaffolding activity, which allows interaction with different complexes with distinct functions, and the ability to guide protein complexes to both close and distant genomic loci confer to these molecules the capability to affect gene expression on a genome-wide scale.

Many lncRNAs involved in epigenetic regulation are thought to interact with the polycomb repressive complex 2 (PRC2), which deposits the repressive histone 3 Lys 27 trimethylation (H3K27m3) histone mark, in order to repress gene transcription. However, different studies have shown that PRC2 binds unspecifically to any RNAs [50,51], raising the important question of the relevance of this interaction in lncRNA function.

### 2.1. Xist

One of the most studied lncRNA is the X-inactive specific transcript (Xist), which is involved in the initial phase of X chromosome inactivation (XCI) in early female embryonic development. Xist represents the first examples of lncRNA directly involved in the formation of repressive chromatin [52]. Early studies defined that Xist, through specific RNA regions, coordinates the tethering of chromatin modifiers to one of the two X chromosomes allowing transcriptional silencing [53,54]. The PRC2 complex was initially described as direct interactor of Xist [55,56,57]. However, recent biochemical purification approaches combined with functional studies identified two proteins that directly bind Xist and seem to be required for PRC2 and SMRT/HDAC3 recruitment: hnRNPK (heterogeneous nuclear ribonucleoprotein K) and SHARP (SMRT/histone deacetylase 1 (HDAC1)-associated repressor protein) [58,59]. Notably, while SHARP and the associated SMRT/HDAC3 are required for initiation of X inactivation, PCR2 is dispensable at this stage [58,59]. PCR2 is instead needed for the maintenance of transcriptional silencing during the imprinted phase of X-chromosome inactivation [50].

Aberrant expression of Xist and X-chromosome overexpression was found in different types of human cancers. The importance of dosage compensation of X-linked genes is reasoned by the presence of many potential tumor-suppressor or cancer-promoting genes on X chromosome [60]. Indeed, human malignancies frequently show X aneuploidy [60] and female mice carrying Xist deletion in the hematopoietic compartment develop an aggressive myeloproliferative disorder with full penetrance [49]. A paradigm for the link between X-chromosome inactivation defects and cancer is the *BRCA1* gene, a tumor-suppressor gene that is mutated in 80–90% of inherited breast-ovarian cancer syndrome [61]. Indeed, beyond its role in DNA repair and genomic instability [62,63], *BRCA1* has been shown to be involved in X-chromosome inactivation. In particular, it was described to colocalise with Xist supporting its accumulation on the inactive X chromosome [64]. Notably, perturbation of X-chromosome inactivation with loss of the silenced state is observed in *BRCA1*-deficient condition. On the contrary cells from sporadic breast and ovarian cancers, which do not carry mutations of *BRCA1*, show proper hallmarks of X-chromosome inactivation [65].

### 2.2. HOTAIR

Besides Xist, other cancer-associated lncRNAs have been reported to interact with PRC2 and other chromatin regulatory complexes. HOX transcript antisense RNA (HOTAIR) is a lncRNA transcribed form the *HOX-C* cluster on chromosome 12 and it has been shown to regulate *in trans* the expression of *HOXD* genes on chromosome 2 [26]. In addition to regulate *HOX* genes, HOTAIR can contribute to the epigenetic repression of several genes within our genome and its misregulation was shown to contribute the epigenetic alterations of different type of cancer cells promoting tumor growth and metastasis [27,28,29,30]. This effect was related to its ability to adopt a defined secondary structure through which it associates with and coordinates the chromatin modifying activities of PCR2, which deposits the repressive H3K27m3 marks, and LSD1-CoREST-REST complex, which inhibits transcription trough the demethylation of trimethylated histone 3 Lys 4 (H3K4m3) [29,31]. In particular, HOTAIR is strongly upregulated in primary breast tumors and breast cancer metastases and overexpression studies performed in a cell line model of breast cancer have shown that elevated HOTAIR levels resulted in the relocalization of PRC2 to several hundred genes [29]. However, a more recent study showed that the repressing activity of HOTAIR in breast cancer cells does not require PRC2 and that the recruitment of this complex is instead a consequence of gene silencing that is established by the lncRNA with a still not defined mechanism [32].

### 2.3. Other Polycomb Repressive Complexes Regulators

In addition to HOTAIR, other lncRNAs, such as neuroblastoma associated transcript 1 (NBAT1, also known as CASC14), LINC-PINT (also known as MKLN1-AS1) and MIR31 host gene (*MIR31HG*) have been shown to interact with PRC2 to influence the epigenetic state of cancer cells. However, it should be noted that the significance of the interaction between PRC2 and lncRNA is currently under active debate.

NBAT and LINC-PINT are examples of PRC2 regulators that act as tumor suppressor genes. Initially, decreased expression of NBAT1 was initially associated with poor patient prognosis in neuroblastomas. Loss of NBAT1 promotes proliferation and impairs differentiation of neuronal precursors [43]. Interestingly, NBAT1 interaction with PRC2 controls tumor progression by suppressing oncogenes such as SOX9, VCAN, and OSMR. On the other hand, NBAT-1 promotes neuronal lineage commitment by suppressing NRSF/REST by interacting with an unknown neuronal-linage-specific transcriptional repressor. The PRC2 interaction is not involved in the repression of NRSF/REST [41]. Later on, NBAT1 was found downregulated in various types of cancer [44]. In particular, reduced NBAT1 in breast cancer is associated with tumor metastasis and poor clinical outcome. The effect of NBAT1 in breast cancer is mediated, at least in part, through DKK1, an inhibitor of Wnt (Wingless-related integration site) signaling pathway. However, in this case, the interaction of NBAT1 with PRC2 is not required to guide but to repress its catalytic function. This activity is consistent with a recent model in which RNA and chromatin compete for PRC2 binding. Moreover, RNA not only prevents PRC2 recruitment to chromatin at active genes but also inhibits its catalytic activity [66].

Similarly, LINC-PINT results downregulated in different types of tumors [36,37] and its expression decreases even more with the progression of the disease or with the acquisition of an aggressive phenotype in tumor xenograft models [38]. LINC-PINT is conserved among vertebrates and its expression is induced by the oncosuppressor p53 both in human and in mouse [36]. It has been suggested that the activity of LINC-PINT is dependent on a highly conserved sequence element that interacts with PRC2 and is required for the PRC2-dependend silencing of genes associated with cell invasion [37].

MIR31HG regulates the Oncogene-induced senescence (OIS), an important tumor suppressor mechanism, by repressing the expression of the *INK4A* gene (encoding the p16 tumor suppressor) [42,67]. In melanoma, the expression of MIR31HG is inversely correlated to p16^Ink4a^ mRNA. Also in this case, the epigenetic repression of the *INK4A* locus is mediated by the interaction of MIR31HG with PRC2 [42].

HOXA11-AS is another cancer-related lncRNA that with its scaffold activity is able to interact using different structural domains with the chromatin modification factors PRC2, LSD1, and the DNA methyltransferase DNMT1 [33]. HOXA11-AS is upregulated in gastric cancer where it promotes cancer cell proliferation and migration. It has been suggested that it exerts its oncogenic function by guiding the chromatin modification factors to specific genes; among them Sun and colleagues identified two novel tumor suppressor genes, *PRSS8* and *KLF2*, which are involved in cancer cell proliferation, apoptosis, and invasion. The expression of these genes in gastric cancer inversely correlates with the expression of HOXA11-AS, which is required for their silencing favouring the binding and activity of PRC2, LSD1, and DNMT1 on their promoters [33]. However other studies indicated that HOXA11-AS could also have also a tumor suppressor function; for instance Li et al. showed that HOXA11-AS resulted downregulated in colorectal cancer (CRC) tissues and thus is associated with a poor prognosis. Altogether these findings suggest that HOXA11-AS may act as oncogene or tumor suppressor depending on the cellular context [34].

ANRIL (also known as CDKN2B-AS1) is an antisense lncRNA overlapping the *INK4B-ARF-INK4A* locus. The latter encodes for three tumor-suppressor genes: *INK4B*, *ARF* and *INK4A*. These genes have a key role in oncogene-induced senescence and results upregulated during premalignant lesion limiting tumor progression [67]. Therefore, it is not surprising that both deletion of the entire the *INK4B-ARF-INK4A* locus and inactivation of each single gene due to mutations or aberrant epigenetic modifications are recurrent across tumors and cancer-cell lines [68,69,70]. Under normal conditions, the lncRNA ANRIL acts as a platform for the recruitment of both PCR1 (via CBX7 protein) and PCR2 (via Suz12 protein) complexes on the *INK4B-ARF-INK4A* locus helping to initiate and maintain its silenced state [8,9]. However, the locus retains the possibility to undergo chromatin reorganization in order to reactivate its expression when needed. Notably, this dynamic regulation failed in many neoplastic transformations such as in neoplastic epithelial tissues [9], oesophageal squamous cell carcinoma [10] and leukaemia leukocytes [11,12] where ANRIL overexpressed and as a consequence, a most robust and stable silencing of the *INK4B-ARF-INK4A* locus occurs.

Another important locus that is epigenetically controlled in cancer by the activity of lncRNAs is that one encoding for the negative cell-cycle regulator *CDKN1A* (encoding the p21 tumor suppressor). LED (lncRNA activator of enhancer domains) and FAL1 (focally amplified lncRNA on chromosome 1) lncRNAs are able to specifically control the expression of *CDKN1A* gene, even though through different mechanisms, and their deregulation in cancer accounts for the loss of p21 activation. In particular, LED is an enhancer RNA (eRNA), induced by p53, that interacts with an enhancer region of *CDKN1A* gene and promotes its transcription by favoring histone 3 Lys 9 acetylation (H3K9Ac) [35]; on the contrary, FAL1 (focally amplified lncRNA on chromosome 1) acts as a repressor of *CDKN1A* gene by recruiting the chromatin repressing the ring-finger protein BMI1, a component of PRC1 [18]. Notably, while LED is silenced in a subset of p53 wild-type leukaemia cells, indicating a tumor-suppressing role, FAL1 has an oncogenic activity since is located in a region of chromosome 1, which is frequently amplified in cancer. However, both conditions result in the suppression of p21 expression, thus promoting tumor cell proliferation. CRNDE (colorectal neoplasia differentially expressed) is another lncRNA that regulates *CDKN1A* expression at the epigenetic level. In colorectal cancer (CRC) tissues CRNDE resulted upregulated and its expression levels positively correlates with advanced pathological stages and large tumor size. Suppression of CRNDE expression in CRC cells inhibits cell proliferation and results in the upregulation of the tumor suppressors *DUAP5* and *CDKN1A*. Similarly to HOTAIR, it has been demonstrated that CRNDE interacts with PCR2 via EZH2 and contributes to establish a silenced chromatin state on promoters of the two genes, therein promoting tumor development [71].

## 3. Regulators of DNA Methylation

DNA methylation is one of the major forms of epigenetic regulation of gene expression that accompanies development and cell differentiation [72,73]; it consists in a covalent addition of a methyl group to cytosines that are usually restricted to CpG dinucleotides [74]. Promoter and first-exon regions often contain CpG islands and when these undergo methylation the transcription of the associated gene is repressed [75]. In mammalian, the DNA methylation is achieved by three distinct S-adenosylmethionine (SAM)-dependent DNA methyltransferases: DNMT3a and DMNT3b involved in de novo DNA methylation and DNMT1 actin in the maintenance of the methylation status during DNA replication [74]. Abnormal methylation patters have been reported in many types of tumors where a global hypo methylation is combined with a hyper methylation occurring on specific regions. While most of the hypo methylation events occur on repetitive elements, resulting in activation of transposable elements and increased genomic instability, the hyper methylation is more frequent on promoter-associated CpG islands, often associated to key tumor suppressor genes [76,77].

Different lncRNAs can regulate the methylation status of DNA in human cell by recruiting or inhibiting the action of DNA methyltransferases and demethylases (Figure 1). These lncRNAs, when aberrantly expressed, may contribute to the aberrant DNA methylation occurring in both CpG islands and CpG island shores [78], which characterizes different cancers.

### 3.1. H19

H19 is a maternal imprinted lncRNA that is highly express during embryogenesis and rapidly downregulated in most tissues after birth [79]. However, it can be reactivated during adult tissue regeneration and tumorigenesis. Even though initially described as tumor suppressor gene because its transcription competes with that of the nearby oncogene *IGF2* [80], in recent years, it has become clear that H19 itself behaves like an oncogene [81,82,83]. H19 expression is mostly regulated by DNA methylation occurring on the DMD region (Differentially Methylated Domain) [19] however it can also be modulated by factors that play a critical role in tumorigenesis: for instance, it can be activated by the oncogenic factor c-Myc [23] or repressed by the tumor suppressor p53 [25]. In accordance with this, H19 resulted highly expressed in primary breast and lung cancer biopsies where c-Myc was upregulated, in p53 null mice prior to tumor development and upon hypoxia in p53 mutated carcinoma cells [22,23,24]. The oncogenic-like activity of this lncRNA is also supported by the decrease of the tumorigenic phenotype observed for a panel of breast and lung cancer cell lines upon H19 knock down [23]. It has been reported that H19 is able to cause a broad DNA methylation changes by direct interaction and inhibition of the S-adenosylhomocysteine hydrolase (SAHH). This enzyme hydrolyses the S-adenosylhomocysteine (SAH), a by-product of transmethylation reactions, which is a potent inhibitor of SAM-dependent methyltransferases. Indeed, the indirect inhibition of DNMT3B activity by H19 is responsible for the loss of methylation at numerous genomic loci and allows spurious transcription in endometrial cancer cells [19].

### 3.2. DACOR1 and ecCEBPA

LncRNA DNMT1-associated colon cancer repressed lncRNA 1 (DACOR1) emerged as an intriguing candidate from a large RNA immunoprecipitation screening performed in colon cancer cell line HCT116 with the aim to identify DMNT1 interacting lncRNAs [15]. DACOR1 is highly and specifically expressed in colon tissue and resulted downregulated in colon tumors and patient-derived colon cancer cell lines [15]. Notably rescuing the level of DACOR1 in these cell lines resulted in reduced clonogenic potential possibly by modulating several cellular pathways. For instance, DACOR1 represses the expression of different genes that inhibit TGF-β/BMP signalling, which has tumor suppressor activity in the colon [84,85]. In addition, DACOR1 downregulates several genes involved in de novo serine biosynthesis thus attenuating the pyruvate kinase M2 (PKM2) activity, which requires serine [86]. Notably, PKM2 has been recently shown to be a key gene in cancer metabolism [87].

DACOR1 is able to associate to chromatin at specific genomic loci and, intriguingly, 20% of them match the position of regions differently methylated in colon cancer samples respect to normal tissues. Therefore, it has been proposed that DACOR1 by interacting with both chromatin and DNMT1, is able to guide DNMT1 protein complex to particular genomic loci thus regulating the expression of specific genes. In particular, among genes repressed by this lncRNA, there is the Cystathionine β-synthase (CBS). CBS downregulation leads to an increase of methionine used to produce SAM, the key methyl donor for DNA methylation suggesting that DACOR1-DMNT1 activity also might impinge on genome wide DNA methylation by regulating cellular SAM levels [15].

Another interesting lncRNA that directly interacts with DNMT1 is the extra-coding CEBPA (ecCEBPA) [16]. However, differently from DACR1, it inhibits DNMT1 acting as a competing molecule thus counteracting DNA methylation. ecCEBPA transcript encompasses the *CEBPA* coding gene in the same sense orientation and shares a concordant expression pattern with CEBPA mRNA in human tissues. Notably, depletion of ecCEBPA leads to a decrease of CEBPA expression and this correlates with an increased DNA methylation at the *CEBPA* promoter region. On the contrary, alleviation of methylation intensity with concomitant CEBPA expression has been observed upon ecCEBPA overexpression. The mechanism of ecCEBPA action proposed relies on two specific regions of the lncRNA: one is able to form locus-specific triplex/quadruplex structure [88] and the other is capable to adopt a stem-loop-like structure for interacting and inhibiting DNMT1 [16]. It has been reported that ecCEBPA levels are upregulated in gastric cancer tissues compare to noncancer ones [17], suggesting that, as consequence, also *CEBPA* gene transcription increases; indeed, one of the CEBPA target genes, the lncRNA UCA1 [89,90], is upregulated in gastric cancer tissues. Notably, higher expression of UCA1 is associated with tumor grades, types and stages [89].

### 3.3. TARID

DNA methylation is a reversible modification that can be erased either by inhibition of methyltransferases or by active enzymatic reactions [91]. The lncRNA TARID (TCF21 antisense RNA inducing demethylation) is able to positively control the expression of the TCF21 gene by inducing active promoter demethylation. TCF21 is a basic helix-loop-helix transcription factor, important for mesenchymal to epithelial transitions [46] and acting as a tumor suppressor gene; in fact, it is frequently silenced in human cancers by aberrant hypermethylation in its promoter region [47,48,92]. It has been reported that the lncRNA TARID is required to maintain the TCF21 promoter region in an open/demethylated state through the interaction with stress response protein GADD45. This latter is able to recruit components of the DNA repair complexes and to lead to site-specific replacement of methylated cytosines by unmethylated ones [93,94]. Notably, few CpG dinucleotides at the transcription start site TCF21 are subjected to demethylation reaction; Arab and coworkers have shown that such specificity is achieved by the ability of TARID to act as a scaffold to bring *GADD45* and *TCF21* promoter in close proximity. However, it still not clear whether the interaction between *TARID* and *TCF21* promoter occurs through an RNA:DNA triplex structure [95] or by forming an R-loop, a peculiar structure of CpG island-containing promoters [96].

Since the inactivation of tumor suppressor genes occurs frequently in cancer cells, it can be inferred that TARID, GADD45 and the associated proteins are part of a surveillance mechanism that protects the promoter of tumor suppressor genes from epigenetic silencing via hyper methylation.

## 4. Regulators of Chromosomal Architecture

Beyond site-specific recruitment of histone/DNA modifying factors, some lncRNAs can bind proteins and chromatin to influence chromatin architecture (Figure 1). These lncRNAs activate the transcription of closely located genes by promoting chromatin looping from transcriptional enhancers, such as described for LED (see above), while others interact with regions on different chromosomes allowing the formation of higher-order chromosome architecture that promote long-range interaction between transcription units and regulatory elements. Moreover, by interacting with nucleosome mobilizing complexes these lncRNAs can remodel chromatin to globally control gene expression.

### 4.1. CCAT1-L and LUNAR

LncRNAs transcribed from enhancer regions (eRNAs) are emerging regulatory molecules in cancers since their deregulation is frequently related to aberrant oncogene activation [97,98,99].

The amplification of the 8q24 locus occurs in many types of human cancers. Its oncogenic activity is due to activation of the *MYC* oncogene [100,101,102,103,104,105]. The 8q24 region contains a gene desert with enhancer regions that contact and control *MYC* promoter located several hundred of kilobases trough the formation of chromatin loops in a tissue specific manner [106]. Many lines of evidence point to an implication of lncRNAs originating from this region in *MYC* driven cancers [107,108,109,110]. In particular, a recent work reported that the long isoform of colorectal cancer associated transcript 1 (CAAT1-L, also known as CARLo-5), which is transcribed from 8q24 region and highly expressed in colorectal cancer [13,111], is involved in the formation of regulatory chromatin loops between the *MYC* promoter and its enhancer thus controlling *MYC* expression [14]. Since the overexpression of CAAT1-L activates in cis *MYC* transcription and promotes tumorigenesis, it has been proposed that this lncRNA contributes to the aberrant expression of c-Myc in the pathogenesis of human colorectal cancer [14]. Notably, CAAT1-L downregulation leads to a decrease of MYC transcription and a reduction of the interaction frequencies between two specific enhancer regions and *MYC* promoter. It has been also reported that CAAT1-L interacts with CTCF, a factor able to mediate chromatin looping [112,113,114] and enriched at the MYC promoter, and that knockdown of CCAT1-L also reduces CTCF binding to chromatin. However, how CTCF coordinates with CCAT1-L to regulate the chromatin looping at the MYC locus is still not defined.

Another example of ncRNA behaving as eRNA-like transcript is LUNAR (leukemia-induced noncoding activator RNA). This lncRNA is a downstream target of NOTCH1 signaling, which is aberrantly activated in more than 50% of T cell acute lymphoblastic leukemia (T-ALL) [40,115]. LUNAR results overexpressed in primary T-ALL and expressed at even higher levels in T-ALL samples carrying activating mutations of NOTCH [41]. The expression of LUNAR shows high correlation with its neighbor IGF1R gene (insulin-like growth factor receptor 1) that was previously shown to play an oncogenic role in T-ALL [116]. Genome wide chromosome conformation capture analysis has revealed the presence of a chromatin loop between the promoter region of LUNAR and an enhancer region in the IGF1R locus. Notably, while NOTCH1 occupies this enhancer region and it is able to control LUNAR expression through the chromatin loop, the lncRNA is localized on its own promoter and on the promoter region of IGFR1 and is required for the recruitment of the Mediator complex and the RNA polymerase II on these regions. Indeed, the downregulation of LUNAR leads to a decrease of IGF1R expression and IGF1 pathway activity as well as to growth retardation effects on T-ALL cells [116].

### 4.2. LncTCF7 and SChLAP1

The SWI/SNF complex is an evolutionally conserved complex that mobilizes nucleosomes at gene promoters by using ATP [117]. The SWI/SNF complex associates with transcription factors and well as lncRNAs in order to regulate gene expression [117,118,119]. It has been reported that alteration of SWI/SNF function promotes cancer progression and that somatic inactivation of specific components of SWI/SNF are present in various human cancers [120], suggesting tumor-suppressor activity of this complex. However, through the interaction with different factors in different cellular contexts SWI/SNF factors can also behave as tumor promoters [121].

Two lncRNAs upregulated in cancer cells, lncTCF7 and SChLAP, are able to interact and modulate the activity of the SWI/SNF complex.

LncTCF7 is highly expressed in hepatocellular carcinoma (HCC) and in liver cancer stem cells (CSCs) where it is required for their self-renewal and maintenance [39,121]. Indeed, LncTCF7 downregulation in CSCs leads to a decreased expression of the pluripotent transcription factors Sox2, Nanog and Oct4, reduces tumor initiating capacity upon subcutaneous injection of nude mice and suppresses xenograft tumor growth and tumorigenic cell frequency. Notably, lncTCF7 depletion affects the expression of the nearby TCF7 coding gene as well as many genes belonging to the Wnt signaling pathway; this latter playing a pivotal role in self renewal and differentiation of CSCs [39,122].

It has been reported that lncTCF7 is able to interact with BRG1, BAF170 and SNF5, core components of the SWI/SNF complex, and to recruit these factors to the TCF7 promoter leading to its activation. TCF7 in turn acts as an upstream trigger to initiate Wnt signaling cascade, thus promoting the self-renewal of liver CSCs and hepatic tumorigenesis [122].

On the other hand, in prostate cancer SNF5 acts as tumor suppressor and its activity is impaired by the interaction with SChLAP lncRNA. SChLAP is highly expressed in 25% of prostate cancers and its expression correlates with the metastatic stage, clinical progression and prostate cancer-specific mortality [123]. SChLAP knockdown impairs cell invasion and proliferation in vitro and slower tumor progression in xenografts. Analysing the genes affected by SChLAP depletion showed an inverse correlation with the gene regulated by SWI/SNF, suggesting that SChLAP and the SWI/SNF function in an opposite manner. It has been demonstrated that SChLAP interacts with SNF5 and attenuates its genomic binding to specific loci, thus impairing its ability to regulate gene expression [123].

## 5. Conclusions

Alterations in the epigenetic regulation of genome activity play a critical role in tumorigenesis. The disruption of any factor involved in chromatin modification is likely to have important effects on global gene expression patterns, and we are currently far away from deciphering the precise role nuclear lncRNAs might play in the regulation of the epigenome. Elucidating regulatory networks between lncRNAs and epigenetic factors will provide mechanistic understanding of the interplay between genetic and epigenetic alterations in cancer, and above all, will produce novel strategies for therapeutic intervention. Moreover, regardless of function, lncRNAs might have application as diagnostic and prognostic markers in cancer.

## Figures and Tables

**Table 1:** LncRNAs with an epigenetic function in cancer.

Name	Cancer	Mechanism	Ref.
ANRIL	High expression linked to poor outcome in prostate and gastric cancer.	Interacts with CBX7 and PRC2 to silence the INK4b/ARF/INK4a locus.	[8,9,10,11,12]
CCAT1-L	Upregulated in human colorectal cancers	Regulates long-range chromatin interactions to activate the transcription of the MYC locus.	[13,14]
DACOR1	Downregulated in colon tumors.	Interacts with and inhibits the DNA methyltransferase DNMT1.	[15]
ecCEBPA	Shows inverse correlation with CEBPA in leukaemia cell lines.	Interacts with DNMT1 and prevents CEBPA locus methylation.	[16,17]
FAL1	Frequently amplified in human cancers.	Interacts with PRC1 to silence the CKDN1A locus.	[18]
H19	Promotes oncogenesis in different cancer types.	Interacts with SAHH inhibiting the DNMT3B dependent DNA methylation at different genetic loci.	[19,20,21,22,23,24,25]
HOTAIR	Overexpressed in liver, metastatic breast, lung and pancreatic tumors.	Interacts with PRC2 and LSD1 to silence genes.	[26,27,28,29,30,31,32]
HOXA11-AS	Acts as oncogene or tumor suppressor depending on the cellular context.	Interacts with PRC2, LSD1 and DNMT1 to silence genes	[33,34]
LED	Downregulated in p53 wild-type leukaemia.	Promotes CDKN1A transcription by acting as enhancer RNA.	[35]
LINC-PINT	Downregulated in colorectal cancer	Interacts with PRC2 to silence genes.	[36,37,38]
lncTCF7	Highly expressed in hepatocellular carcinoma and it is a negative prognostic factor in glioma.	Recruits the SWI/SNF complex to activate the expression of the transcription factor TCF7.	[39]
LUNAR1	Upregulated in T-cell acute lymphoblastic leukemia.	Induces chromatin looping and recruits the Mediator complex to activate IGF1R transcription.	[40,41]
MIR31HG	Deregulated in different human cancers.	Interacts with PRC2 to silence the INK4A locus.	[42]
NBAT1	Loss of NBAT1 is asssociated with poor clinical outcome in Neuroblastoma (NB) and breast cancer (BC).	In NB interacts with PRC2 to silence genes while in BC it interacts with PRC2 to repress its activity.	[43,44]
SChLAP1	Overexpressed in a subset of prostate cancers. It is critical for metastasis and predicts poor outcomes.	Inhibits the binding of the SWI/SNF chromatin remodelling to the genome.	[45]
TARID	Deregulated in different human cancers.	Recruits the DNA demethylation regulator growth arrest and DNA damage inducible protein GADD45α to activate the transcription of the tumor suppressor gene TCF21.	[46,47,48]
Xist	Abnormal expression associated with tumor initiation and progression.	Represses gene expression by multiple epigenetic mechanisms.	[49]
